# Wharton's Jelly mesenchymal stem cell‐derived extracellular vesicles induce liver fibrosis‐resolving phenotype in alternatively activated macrophages

**DOI:** 10.1111/jcmm.18507

**Published:** 2024-09-17

**Authors:** Shukoofeh Torabi, Morteza Zarrabi, Faezeh Shekari, Hedie Poorkazem, Majid Lotfinia, Stefan Bencina, Roberto Gramignoli, Moustapha Hassan, Mustapha Najimi, Massoud Vosough

**Affiliations:** ^1^ Department of Regenerative Medicine, Cell Science Research Center Royan Institute for Stem Cell Biology and Technology, ACECR Tehran Iran; ^2^ Department of Tissue Engineering and Regenerative Medicine, School of Advanced Technologies in Medicine Mazandaran University of Medical Sciences Sari Iran; ^3^ Department of Stem Cells and Developmental Biology, Cell Science Research Center Royan Institute for Stem Cell Biology and Technology, ACECR Tehran Iran; ^4^ Physiology Research Center Kashan University of Medical Sciences Kashan Iran; ^5^ Department of Laboratory Medicine, Division of Pathology Karolinska Institutet Stockholm Sweden; ^6^ UOSD Cell Factory IRCCS Istituto Giannina Gaslini Genoa Italy; ^7^ Experimental Cancer Medicine, Institution for Laboratory Medicine and Karolinska University Hospital Karolinska Institute Stockholm Sweden; ^8^ Laboratory of Pediatric Hepatology and Cell Therapy Institute of Experimental and Clinical Research (IREC), UCLouvain Brussels Belgium

**Keywords:** anti‐inflammatory macrophages, extracellular vesicles, liver fibrosis, Wharton's jelly mesenchymal stem cell

## Abstract

The potential of extracellular vesicles (EVs) isolated from mesenchymal stromal cells in guiding macrophages toward anti‐inflammatory immunophenotypes, has been reported in several studies. In our study, we provided experimental evidence of a distinctive effect played by Wharton Jelly mesenchymal stromal cell‐derived EVs (WJ‐EVs) on human macrophages. We particularly analyzed their anti‐inflammatory effects on macrophages by evaluating their interactions with stellate cells, and their protective role in liver fibrosis. A three‐step gradient method was used to isolate monocytes from umbilical cord blood (UCB). Two subpopulations of WJ‐EVs were isolated by high‐speed (20,000 *g*) and differential ultracentrifugation (110,000 *g*). Further to their characterization, they were designated as EV20K and EV110K and incubated at different concentrations with UCB‐derived monocytes for 7 days. Their anti‐fibrotic effect was assessed by studying the differentiation and functional levels of generated macrophages and their potential to modulate the survival and activity of LX2 stellate cells. The EV20K triggers the polarization of UCB‐derived monocytes towards a peculiar M2‐like functional phenotype more effectively than the M‐CSF positive control. The EV20K treated macrophages were characterized by a higher expression of scavenger receptors, increased phagocytic capacity and production level of interleukin‐10 and transforming growth factor‐β. Conditioned medium from those polarized macrophages attenuated the proliferation, contractility and activation of LX2 stellate cells. Our data show that EV20K derived from WJ‐MSCs induces activated macrophages to suppress immune responses and potentially play a protective role in the pathogenesis of liver fibrosis by directly inhibiting HSC’s activation.

## 1 INTRODUCTION

The cellular and molecular basis of liver fibrosis is a highly conserved wound‐healing response to chronic liver injury, characterized by the accumulation of extracellular matrix components in liver tissue, ultimately leading to organ dysfunction.^1^ Previous studies described the dynamic changes in hepatic macrophage populations during the development and resolution of liver fibrosis.^2^, ^3^ During fibrogenesis, macrophages are recruited to the liver and polarized into proinflammatory phenotypes in response to the local microenvironment cues. such proinflammatory M1 macrophages promote fibrosis by secreting inflammatory cytokines and inducing the activation of hepatic stellate cells (HSCs), responsible for extracellular matrix overproduction. Conversely, in the fibrotic resolution phase, the tissue‐resident macrophages peak and acquire an anti‐inflammatory M2 phenotype, promoting HSCs apoptosis and extracellular matrix degradation. Such a switch in macrophage phenotype is mediated by the phagocytosis of cellular debris and secretion of anti‐inflammatory cytokines in the surrounding microenvironment.^4^, ^5^, ^6^ Consequently, the macrophage phenotype switch has important implications in the liver fibrosis regression and serves as a ground for the development of novel innovative therapeutic strategies. Indeed, several studies highlighted the potential of anti‐inflammatory macrophage as a promising cell‐based therapy for the treatment of liver fibrosis.^7^, ^8^ Administration of mature macrophages into a carbon tetrachloride (CCL4)‐induced murine liver injury model, ameliorated liver fibrosis by stimulating intrinsic liver regeneration, promoting the recruitment of monocytes and neutrophils via chemokine gradients inhibiting the activation of myofibroblasts and collagen synthesis.^9^ Intravenous injection of macrophages derived from mouse embryonic stem cells has also revealed effective results in fibrosis regression.^10^ Furthermore, the infusion of human macrophages, pretreated with CSF‐1, into a murine liver fibrosis model was associated with a decrease in liver injury and fibrosis markers and enhanced regeneration.^11^

Mesenchymal stromal cells (MSCs) are adult stem cells isolated from a wide range of adult and perinatal tissues such as bone marrow (BM), adipose tissue (AD), umbilical cord‐derived Wharton's jelly (WJ) and many other tissues which can possess the multipotent differentiation potential and self‐renewal ability. One of the other important characteristics of MSCs is their ability to mediate immunomodulation and tissue regeneration through paracrine mediators vehiculated by soluble factors and extracellular vesicles (EVs). EVs represent a heterogeneous population of exosomes, microvesicles and apoptotic bodies.^12^ Molecular profiling of MSC‐derived EVs has revealed a cargo rich of variety of bioactive molecules, including mRNA, miRNA, cytokines, chemokines and immune‐modulating factors such as TGF‐β1, IL‐10, PTX3, let‐7b‐5p and miR‐21‐5p which are effective in inducing the polarity of macrophages towards an anti‐inflammatory phenotype.^13^, ^14^ This supports the emerging evidences on the critical role of MSCs in liver repair and fibrosis regression which is mediated via modulation of macrophage phenotype.^15^, ^16^ Evaluating the therapeutic potential of MSC‐derived EVs in liver fibrosis has also shown their ability to alleviate liver damage and promote tissue regeneration by reducing the secretion of proinflammatory cytokines, while promoting the proliferation of anti‐inflammatory cells, such as M2 macrophages and Tregs both in vitro and in vivo.^17^, ^18^, ^19^ Compared to MSCs derived from BM or AD, WJ‐MSCs have shown better safety and compatibility, higher proliferation and secretion capacity. Also, WJ‐MSCs produce four times more EVs and secrete anti‐inflammatory factors, including IL‐10, PGE2, IL‐1R and TGF‐β1.^20^, ^21^ WJ‐EVs have been shown to promote the switch of macrophages from M1 into M2‐like states in vitro.^22^, ^23^, ^24^ In a murine model of colitis, injection of WJ‐EVs resulted in a switch in macrophage phenotype towards an M2 state, thereby preventing the progression of the disease.^25^ Considering that MSC‐EVs exhibit unique advantages over recombinant cytokines like CSF‐1 in macrophage polarization,^26^, ^27^ and given our previous report demonstrating the usefulness of this allogeneic source to provide functional macrophages for diagnostic, research purposes, as well as clinical applications,^28^ we evaluated the effectiveness of human WJ‐EVs on macrophages generated from UCB‐derived monocytes.

## 2 MATERIALS AND METHODS

### 2.1 Isolation and characterization of human WJ‐MSCs

Human WJ‐MSCs were obtained from Celltech Pharmed Co. (Tehran, Iran). About 300,000 cells (P4) were cultured in T150 cm^2^ culture flasks (Corning Costar®) until they reached ~80% cell confluency after 6 days. Conditioned medium was collected and then concentrated 10 times using the Viva Flow 50R (Sartorius, VF05H4), resulting in a final volume of 1000 mL. Centrifugation at 3000 *g* for 10 min at 4°C was performed to remove cellular debris from the concentrated medium. The supernatant was then centrifuged at 20,000 *g* (Tomy Suprema 25) for 30 min at 4°C, washed with phosphate‐buffered saline (PBS) (Sigma‐Aldrich) and subjected to a second high‐speed centrifugation to generate EV product labelled as EV20K. In order to isolate a second subset of EVs, the supernatant was ultracentrifuged at 110,000 *g* (Beckman coulter Optimal L‐100XP ultracentrifuge, SW 32 rotor) for 120 min at 4°C. The pellet resuspended in PBS and ultracentrifuged again. The second subset is labelled as EV110K. Both EV subsets (EV20K and EV110K) were reconstituted in PBS and stored at −80°C until use. Western blotting technique was employed to assess the expression of EV‐enriched proteins (CD81, CD63) and the absence of a negative marker calnexin. The ultrastructure of WJ‐EVs was examined using scanning electron microscopy (SEM) (FEI XL‐30 FEG). In order to analyse the size distribution of EVs, dynamic light scattering (DLS) (Malvern, UK) was utilized.

### 2.2 EV uptake and internalization

To quantify their uptake by UCB‐derived monocytes, WJ‐EVs were labelled with calcein AM (Invitrogen, C3099) as previously described.^29^ Briefly, a mixture of 100 μL PBS containing 10 μg of WJ‐EVs and 10 μL of calcein dye (the final concentration 10 μM) was incubated at 37°C for 20 min. The remaining unincorporated calcein dye was then removed using an Amicon centrifugal filter (UFC510096). EVs labelled with calcein were then added to UCB‐derived monocytes for 2 h at 37°C, followed by three washes with PBS to remove unbound vesicles. The intracellular calcium concentration in monocytes was then measured by using flow cytometry to demonstrate the EVs uptake.

### 2.3 Macrophage polarization assay

In order to optimize the type and concentration of WJ‐EVs that would be most effective for macrophage polarization, monocytes were isolated from human UCB according to our previous report.^28^ Royan Stem Cell Technology ethics committee guidelines directed all experiments to be performed with monocytes isolated from at least three healthy donors (Reference: IR.ACECR.ROYAN.REC.1399.056). Isolated monocytes were cultured in a 6‐well ultra‐low attachment plate (Corning Costar®, 3736) at a concentration of 1 × 10^6^ cells/mL of Iscove's Modified Dulbecco's Medium (IMDM) supplemented with 1000 U/mL penicillin and streptomycin, 2 mM GlutaMAXTM, 0.1 mM non‐essential amino acids and 2.5% human Platelet Lysate (hPL; Royan Institute, Iran). Then EV20K and EV110K were added to the culture medium at two different concentrations (10 and 50 μg/mL) and incubated with UCB‐derived monocytes. Positive control (100 ng/mL macrophage colony‐stimulating factor (M‐CSF)) or negative control (50% WJ‐EV‐depleted medium) were used to evaluate the potential specific effect of EVs. The media were changed every 2–3 days until day 7. Thereafter, macrophages were harvested, and mRNA expression levels of genes encoding proinflammatory factors *Interlukin‐6, Tumour necrosis factor alpha, Interleukin 1 beta* (*Il‐6, TNF‐α, IL‐1β*), anti‐inflammatory factors *Interlukin‐10, Transforming growth factor beta* (*Il‐10, TGF‐β*), M2 macrophage‐specific markers *Resistin‐like molecule alpha1, Mannose Receptor C‐Type 1* (*Fizz‐1, MRC1, CD163*), extracellular matrix (ECM) turnover enzymes *matrix Metallopeptidase 1–2, metallopeptidase inhibitor 1* (*MMP1, MMP2, TIMP1*) and pro‐regeneration factors *TNFSF12 TNF superfamily member 12, hepatocyte growth factor* (*TWEAK, HGF*) were evaluated by using real‐time PCR, SYBR Premix Ex Taq II (TaKaRa, RR820) and the ABI StepOnePlus Real‐Time PCR system. The relative transcripts expression levels were normalized to glyceraldehyde‐3‐phosphate dehydrogenase (GAPDH) using the 2^(‐ΔΔCT) method. Table S1 contains the sequences of the PCR primers used.

### 2.4 Flow cytometry characterization

The expression of cell surface markers was evaluated in 3 different groups of mature macrophages [Treated with 100 ng/mL M‐CSF (referred to as M0), treated with 50% WJ‐ EV‐depleted media (M‐EVDM), and treated with 10 μg/mL EV20K (M‐EV20)] by incubating 1 × 10^5^ cells (treated for 7 days as abovementioned) with specific anti‐CD14‐PE (Biolegend, 367104), anti‐CD206‐FITC (Biolegend, 321104), anti‐CD163‐PE (Biolegend, 333606) and anti‐CD86‐PE (BD PharmingenTM, 555658) antibodies for 30 min at 4°C. Following incubation, cells were washed with PBS, and analysed by the BD FACSCalibur Flow Cytometer. To exclude apoptotic or dead cell debris, 7‐amino‐actinomycin D (7‐AAD) (Biolegend, 420403) was used. The results were analysed using FlowJo software (version 7.6; Tree Star Inc., Ashland, OR).

### 2.5 Enzyme‐linked immunosorbent assay

The secreted levels of human TNF‐α, TGF‐β, IL‐6 and IL‐10 were analysed in supernatants recovered at day 7 from cultures of mature macrophages by using enzyme‐linked immunosorbent assay (ELISA) kits (R&D Systems™, D1000B, DTA00D, D6050 and E0134Hu) according to the manufacturer's instructions.

### 2.6 Macrophage phagocytosis assay

The phagocytosis capacity of macrophages was assessed as previously reported.^28^ Briefly, on day 7, mature macrophages were stimulated with or without lipopolysaccharide (LPS) at a concentration of 100 ng/mL for 24 h in a serum‐free medium. We then exposed macrophages to enhanced green fluorescent protein (*EGFP*)*‐expressing E. coli* at a multiplicity of infection (MOI) of 20 for 2 h at 37°C. Subsequently, the cell culture plates were placed on ice for 10 min in order to stop phagocytosis. The macrophages were then incubated with 200 μg/mL gentamicin for 15 min at 37°C in order to kill any remaining extracellular bacteria. Afterwards, the macrophages were washed twice with ice‐cold PBS and internalized fluorescence was detected with a FACS Calibur flow cytometer (BD FACSCaliburTM; BD Biosciences) and fluorescence microscopy (Olympus IX71).

### 2.7 T‐cell proliferation assay

To assess the sustained inhibitory effect of differentiated macrophages on the proliferation of allogenic T‐ cells, peripheral blood mononuclear cells (PBMCs) were isolated from a healthy donor using ficoll gradient centrifugation. The isolated PBMCs were then stained with 5 μM carboxyfluorescein succinimidyl ester (CFSE) for 5 min at room temperature. Thereafter, 1× 10^5^ cell/well were seeded in 96‐well plates (SPL, 34296) containing RPMI medium supplemented with either 10% FBS or 10% pooled serum from rats (*n* = 3) with liver damage induced by CCl_4_. Phytohaemagglutinin (PHA) at 5 mg/mL concentration was also added in the culture medium to induce cell proliferation. Thereafter, M0, M‐EVDM and M‐EV20 macrophages were harvested and added to the wells at a ratio of 1:2 macrophages per T cells. The proliferation rate of T cells was determined using flow cytometry 5 days later, after staining with anti‐CD3 (PE) antibody (Biolegend, 300408) according to the manufacturing protocol.

### 2.8 LX2 stellate cells treatment with macrophages conditioned medium

To evaluate their anti‐fibrotic activity, the conditioned medium (CM) of differentiated macrophages (from the three groups) was incubated with LX2 stellate cells. Such CM is a serum‐free medium recovered after 48 h incubation at 37°C with matured macrophage cultures. After collection, the CM was centrifuged at 3000 *g* for 10 min at 4°C, and stored at −70°C before incubation with LX2 cells.

LX2 cells, obtained from Royan Institute public cell bank, were seeded at a concentration of 5 × 10^4^ cells/cm^2^ in high‐glucose Dulbecco's Modified Eagle Medium (DMEM) supplemented with 2% fetal bovine serum (FBS), 1% penicillin/streptomycin (Pen/Strep), 1% essential amino acids and 1% GlutaMAX in a 6‐well culture plate for 3 days (80% confluence). Differentiation of LX‐2 stellate cells into activated myofibroblasts was induced by treating them with 5 ng/mL of recombinant human TGF‐β1 (R&D Systems) for 2 days.^30^ Then, the supernatant of the LX‐2 cells was replaced with a mixture of 75% fresh DMEM and 25% CM derived from M0, M‐EVDM and M‐EV20 macrophages in the presence of TGF‐β1 for 48 h. LX2 stellate cells were then pelleted, and total RNA was extracted with NucleoSpin RNA Mini kit (MACHEREY‐NAGEL GmbH, Germany) according to the manufacturer's instructions and cDNA was synthesized utilizing the RevertAid H Minus First Strand cDNA Synthesis Kit (Thermo Scientific™, K1632). The expression levels of genes related to stellate cells' activation including *alpha smooth‐muscle actin* (*ACTA2*), *fibronectin* (*FN*), *type I collagen* (*Col1a1*) and *transforming growth factor beta* (*TGF‐β*) were evaluated by RT‐qPCR.

### 2.9 Cell viability (live/dead) assay

LX2 cells were assessed for viability before and after treatment with the CM of differentiated macrophages using the Live/Dead assay. LX2 cells were seeded at 5 × 10^4^ cell/cm^2^ in a 24‐well culture plate until reaching 70% confluency. LX2 stellate cells differentiated or not after 48 h treatment with TGF‐β were incubated with a mixture of 75% fresh DMEM and 25% CM obtained from macrophages M0, M‐EVDM and M‐EV20. Following 48 h of incubation, the LX2 stellate cells were stained using the Live/Dead assay kit (Invitrogen R37601) and images were taken using a fluorescence microscope (Olympus IX71).

### 2.10 Annexin V/PI assay for apoptosis

Annexin V‐fluorescein isothiocyanate (FITC)/propidium iodide (PI) apoptosis kit was utilized following the manufacturer's protocol. LX2 cells were seeded at a concentration of 4 × 10^4^ cells/cm^2^ in a 6‐well culture plate. As described above, once the cells have reached a confluency of 70%, they were differentiated into myofibroblasts and incubated with macrophage CM. Following 48 h of incubation, the LX2 cells were washed with PBS, resuspended in 100 μL of 1X Annexin V binding buffer and then incubated with 5 μL of Annexin V‐FITC and 5 μL of PI for 20 min at room temperature in the dark. After that, the levels of apoptotic cells were analysed by using a FACS Calibur flow cytometer (BD FACSCaliburTM; BD Biosciences).

### 2.11 Collagen gel contraction assay

In order to assess the effects of macrophage CM on the contractile activity of LX2 cells, a collagen gel contraction assay was conducted as previously described.^31^ Type I collagen was extracted from rat tail tendons and processed in 0.05 M acetic acid solution to obtain sterile soluble collagen. Then, LX2 stellate cells (undifferentiated and differentiated after 48 h incubation with TGF‐β) were suspended in 500 mL of DMEM containing 3 mg/mL soluble collagen at a concentration of 1 × 10^6^ cells/mL. The mixture was then added into a 24‐well tissue culture plate and incubated for 1 h at 37°C to polymerize the gel. Thereafter, a mixture of 50% fresh DMEM (with and without TGF‐β) and 50% CM from M0, M‐EVDM and M‐EV20 macrophages was added to the top of each gel layer. Images of the collagen gels were taken after 48 h and Image J software was used to assess the degree of contraction.

### 2.12 Statistical analyses

Statistical analysis was performed using GraphPad Prism9, and the results are presented as means ± SD. Significant differences were assessed using a two‐tailed Student's *t*‐test or two‐way ANOVA with Tukey's post hoc test (*p ≤ 0.05, **p ≤ 0.01, ***p ≤ 0.001).

## 3 RESULTS

### 3.1 Characterization of WJ‐MSC‐derived EVs

**FIGURE 1:**
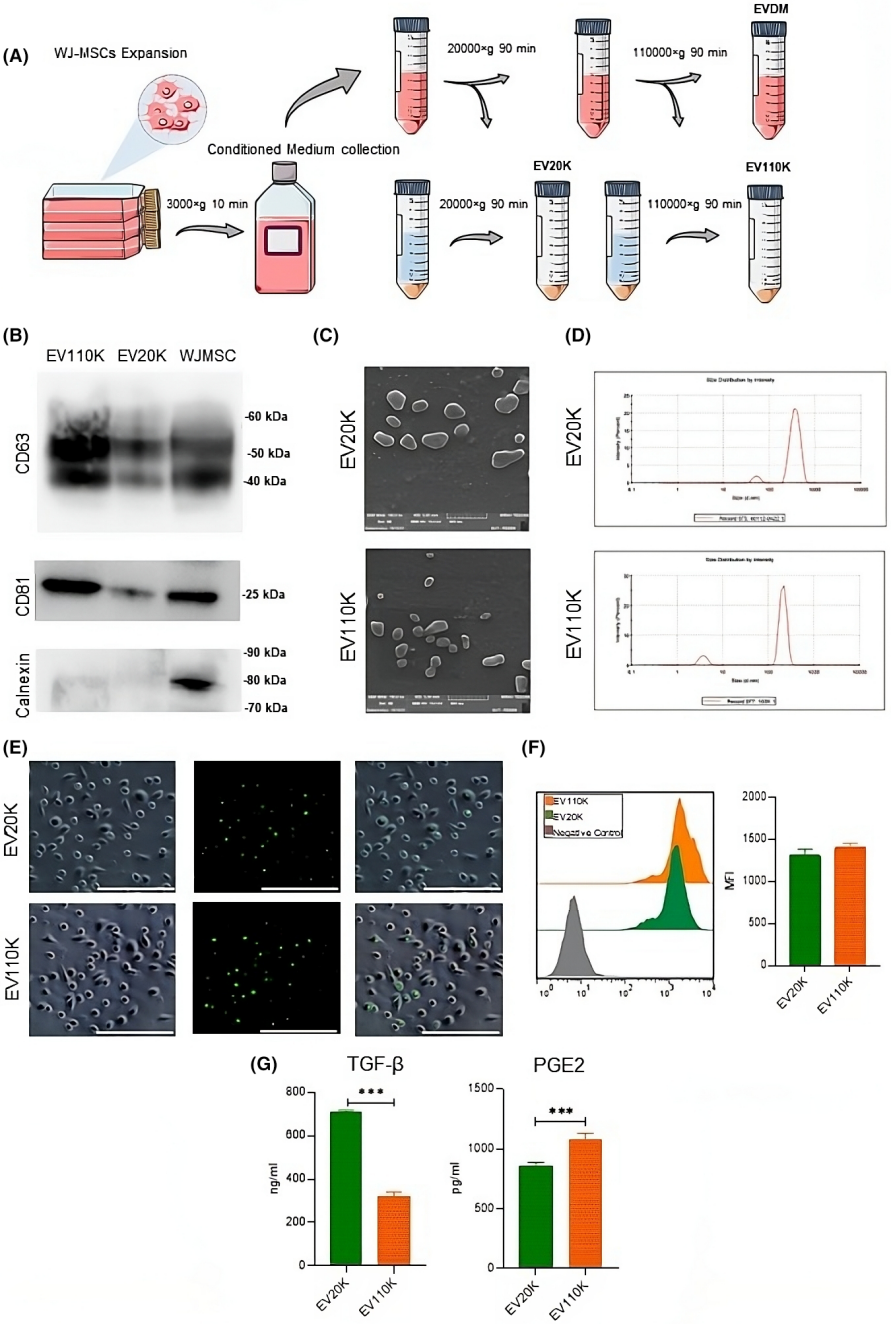
Isolation, characterization and tracking of WJ‐MSC‐EVs. (A) Schematic protocol for the isolation of WJ‐MSC‐EVs. To remove debris, conditioned medium (CM) obtained from WJ‐MSCs was centrifuged at 300 *g* for 20 min and concentrated using viva flow. Afterward, the supernatant was centrifuged at 20,000 *g* for 20 min. Following centrifugation with PBS at 20,000 *g* for 30 min, the pellet containing EV20K was separated. EV110K was isolated by ultracentrifuging the supernatant obtained from the previous step for 120 min at 110,000 *g*, discarding the supernatant and ultracentrifuging the pellet obtained in this step for 120 min at 110,000 *g*. (B) Western blot analysis revealed expression of the specific EV markers CD63, CD81, by EV20K and EV110K isolated from the CM of WJ‐MSCs. However, the negative control calnexin was not expressed. (C) SEM images of EV20K and EV110K showing their spheroidal morphology (scale = 500 nm). (D) EV20K had an average diameter of 230 nm and EV110K had an average diameter of 195 nm, based on DLS size distribution data. (E, F) Clennexin expression indicates the entry of WJ‐EVs into monocytes based on flow cytometry and florescent microscopic data. (G) ELISA was used to evaluate the expression of M2‐inducing factors in two WJ‐MSC‐EVs populations. DLS, dynamic light scattering; EV110K, extracellular vesicles isolated by 110,000 *g*; EV20K, extracellular vesicles isolated by 20,000 *g*; PBS, phosphate‐buffered saline; SEM, scanning electron microscopy; WJ‐MSC‐EVs, Wharton Jelly MSC extracellular vesicles. ***p < 0.001.

EV20K and EV110K were isolated from the CM of WJ‐MSCs using a series of differential high‐speed centrifugation steps followed by ultracentrifugation (Figure 1A). Based on western blot analysis, we confirmed the expression profile of both EV subsets thanks to their immunopositivity for specific EV markers CD63 and CD81, but not for the non‐EV marker calnexin (Figure 1B). Morphological aspect was confirmed by SEM analysis, as both EV subsets characterized by spheroidal shape and integrity (Figure 1C). Size distribution analysis based on a dynamic light scattering (DLS) also indicated that dimensions of EV20K and EV110K are of approximately 230 and 195 nm, respectively (Figure 1D). Thereafter, we demonstrated their efficient uptake by UCB monocytes as revealed by fluorescent localization into monocyte cytoplasm, within 2 h of incubation at 37°C (Figure 1E). Both EV20K and EV110K presented similar fluorescent intensity once internalized (Figure 1F). Finally, a proteomic examination of the content of EVs revealed a significant amount of anti‐inflammatory proteins (PGE2 and TGF‐β) in the EV cargos, with 3 times more TGF‐β in EV20K compared to EV110K and less PGE2 (Figure 1G).

### 3.2 EV20K effectively induces the generation of anti‐inflammatory macrophages

**FIGURE 2:**
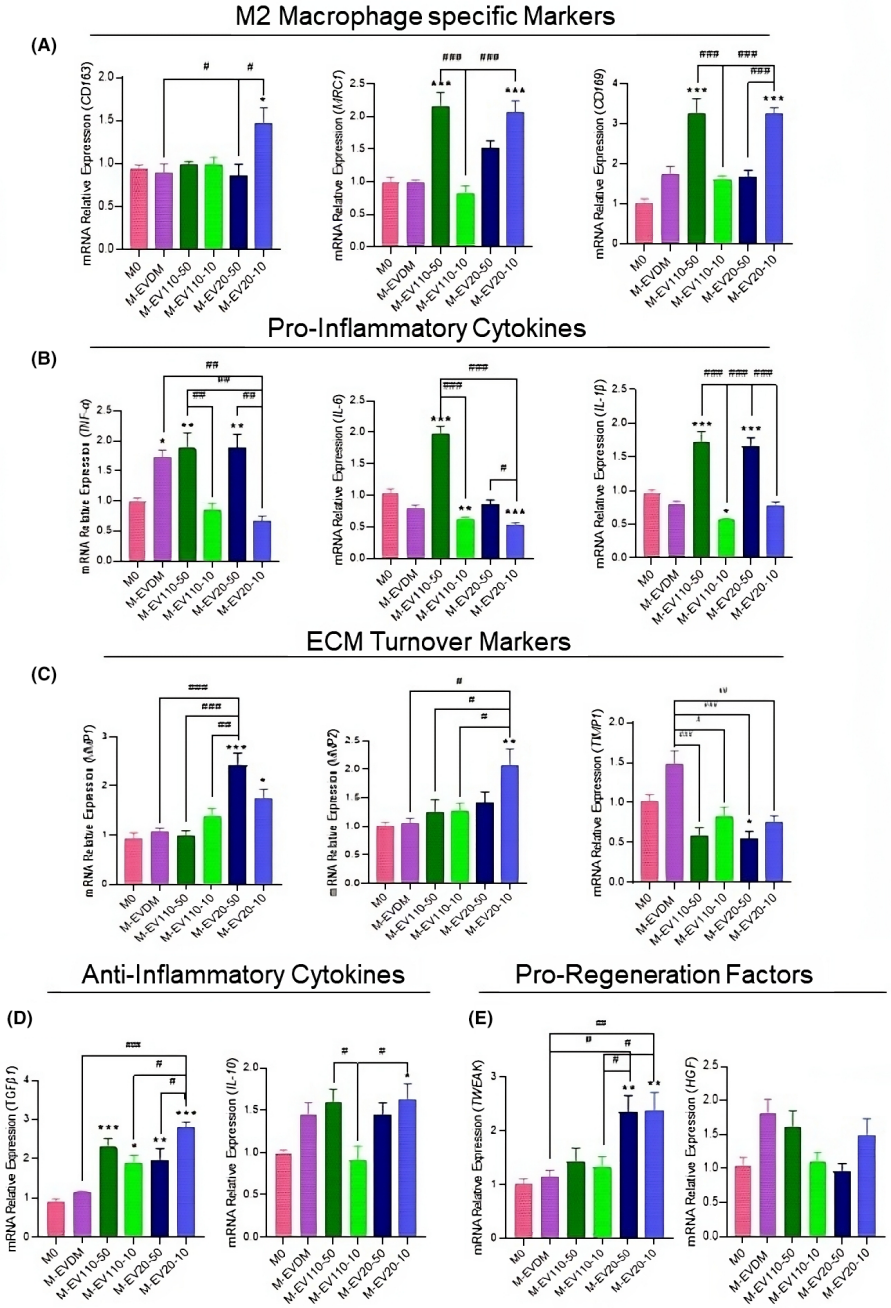
Real‐time PCR analysis of gene expression in M0, M‐EVDM and M‐EV20 macrophages. Real‐time PCR was used to determine gene expression changes in differentiated macrophages, M0 (control cells treated with 100 ng/mL M‐CSF), EVDM (cells treated with 50% EV‐depleted media) and M‐EV100‐10/50 and M‐EV20‐10/50 treatment with different concentrations (10 to 50 μg/mL) of EV110K andEV20K. Column bars represent the mean ± SD of three independent experiments with macrophages from three different healthy donors. Statistically significant differences using One‐way analysis of variance followed by Tukey's post hoc test (*p ≤ 0.05, **p ≤ 0.01, ***p ≤ 0.001) compared to M0 group (^#^ *p* ≤ 0.05, ^##^ *p* ≤ 0.01, ^###^ *p* ≤ 0.001). EVDM, EV‐depleted medium; M‐CSF, macrophage colony‐stimulating factor.

We further investigated the effects of WJ‐MSC‐EVs on the polarization of UCB monocytes, with particular attention to cell fate switch into anti‐inflammatory and anti‐fibrotic phenotypes. UCB moncoytes were incubated with macrophage colony‐stimulating factor (M‐CSF), EV‐depleted medium (EVDM) and two subsets of EV20K and EV110K at both (10 and 50 μg/mL) for 7 days. Six different macrophage preparations: M0 (Control), M‐EVDM, M‐EV110‐50, M‐EV110‐10, M‐EV20‐50 and M‐EV20‐10 were collected and further analysed by RT‐qPCR. From the data recovered, the transcriptome expression levels of M2 macrophage‐specific markers (*MRC1, CD163, CD169*), and anti‐inflammatory cytokines (*IL‐10 and TGF‐β*) were induced in the macrophages treated with 10 μg/mL EV20K (M‐EV20‐10) and were associated to lower expression of proinflammatory (*IL‐6, IL‐1β, TNF‐α*) and pro‐fibrotic (*TIMP1*) markers in comparison to M0 and M‐EVDM treated groups (Figure 2B,C). Moreover, there was an increase in the expression of anti‐fibrotic markers (*MMP‐1* and *‐2*) and pro‐regenerative factors (*TWEAK* and *HGF*) (Figure 2A–E) in this group. Concurrently, when macrophages were treated with 50 μg/mL EV110K (M‐EV110‐50), increased expression levels of M2 macrophage‐specific markers (*CD206, CD169*) and anti‐inflammatory cytokines (*IL‐10* and *TGF‐β*) were observed (Figure 2A,D). However, proinflammatory genes (*IL‐6, IL‐1β, TNF‐α*) were also significantly upregulated in this group (Figure 2B), which were consistent with our previous research, revealing that MSCs derived EV110K had a higher quantity of proteins involved in inflammatory responses than EV20K (under editorial decision). Hence, because EV20K showed a potent anti‐inflammatory and anti‐fibrotic effects on treated macrophages at lower concentrations in comparison to EV110K and because the isolation of EV20K is more practical, feasible, cost‐effective and aligned with good manufacturing practices (GMP), we decided to use EV20K subset for our further investigations (Figure 2).

### 3.3 EV20K‐induced expression of M2 markers and phagocytic abilities of macrophages

**FIGURE 3:**
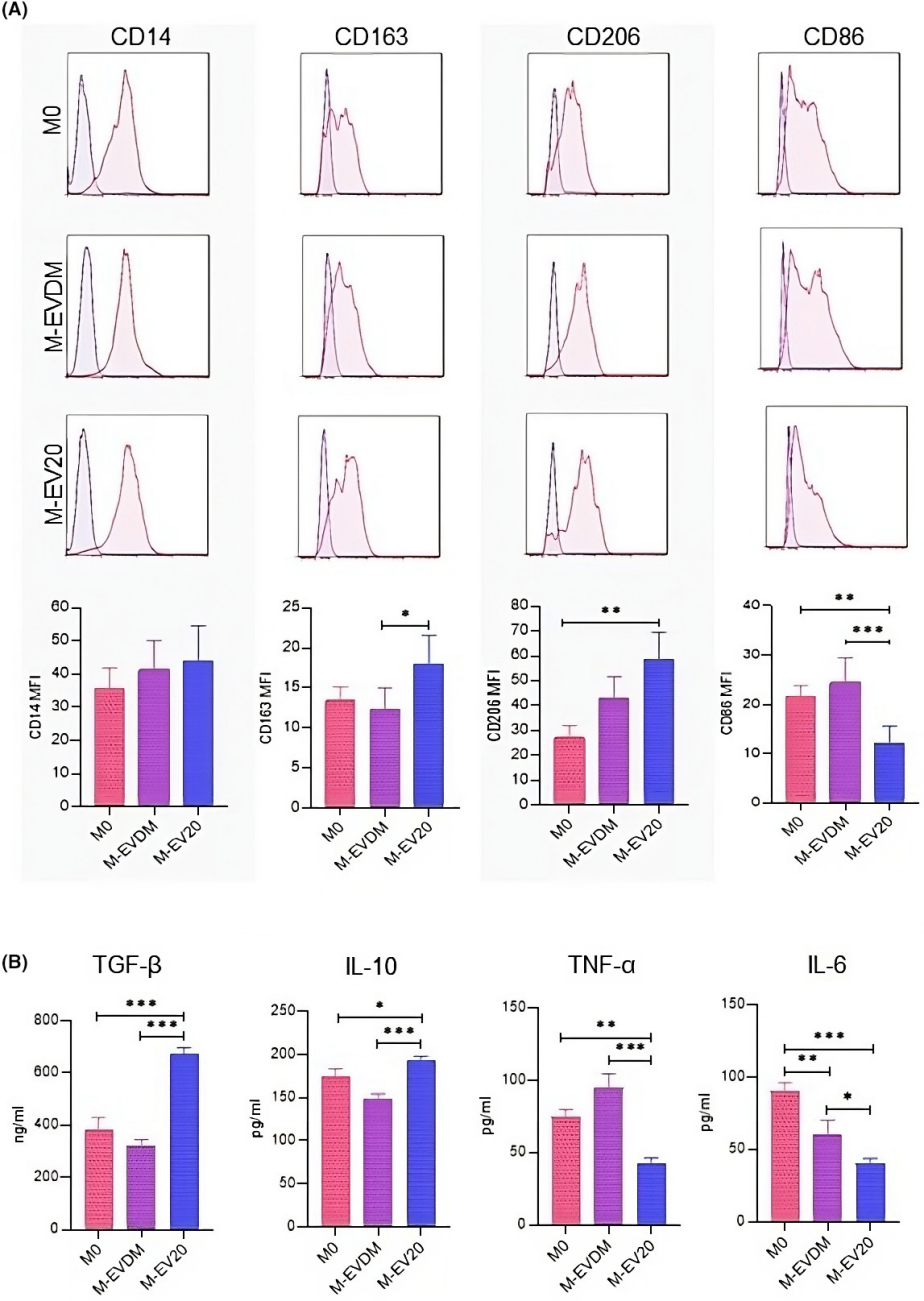
Surface marker expression pattern of differentiated macrophages. M0, M‐EVDM and M‐EV20 macrophages were generated after 7 days as described above. Data are expressed as mean ± SD of percentages of M2 and M1 surface markers from three independent experiments with macrophages from three different healthy donors. Statistically significant differences were determined by One‐way ANOVA followed by Tukey's post hoc test (*p ≤ 0.05, **p ≤ 0.01, ***p ≤ 0.001).

In order to confirm the anti‐inflammatory phenotype induced by EV20K, we analysed the expression of specific cell surface markers on differentiated M‐EV20 macrophages by using flow cytometry. Phenotypic analysis based on mean fluorescence intensity (MFI) showed an increased expression of CD206 (MFI ratio: 27.17 ± 4.97 vs 42.82 ± 8.97 vs 58.52 ± 10.8; *p* ≤ 0.01), CD14 (MFI ratio: 35.72 ± 6.04 vs 41.25 ± 8.83 vs 43.9 ± 10.6; *p* ≤ 0.05) and CD163 (MFI ratio: 13.42 ± 1.75 vs 12.31 ± 2.6 vs 17.9 ± 3.6; *p* ≤ 0.05) in those macrophages having been incubated with EV20K at 10 μg/mL in comparison to M0 and M‐EVDM macrophages. Conversely, CD86 (MFI ratio: 21.64 ± 2.10 vs 24.44 ± 5 vs 11.92 ± 3.6; *p* ≤ 0.001), a specific marker for M1 macrophages, was detected at lower expression in this group (Figure 3A). Proteomic analyses of the supernatant from differentiated macrophage cultures also revealed a higher expression of anti‐inflammatory cytokines TGF‐β (M0: 380.51 ± 47.6 vs M‐EVDM: 317.48 ± 27.7 vs M‐EV20: 675.11 ± 22.1; *p* ≤ 0.001) and IL‐10 (M0: 173.86 ± 9.54 vs M‐EVDM: 149 ± 5.49 vs M‐EV20: 193.43 ± 4.10; *p* ≤ 0.001), and a significantly lower amount of proinflammatory cytokines IL‐6 (M0: 90.27 ± 5.74 vs M‐EVDM: 59.64 ± 10.6 vs M‐EV20: 40.06 ± 3.50; *p* ≤ 0.001) and TNF‐α (M0: 47.72 ± 5.10 vs M‐EVDM: 94.66 ± 9.71 vs M‐EV20: 41.91 ± 4.21; *p* ≤ 0.001) in M‐EV20 macrophages in comparison to both other groups (Figure 3B).

### 3.4 EV20K enhances the phagocytic activity of macrophages

**FIGURE 4:**
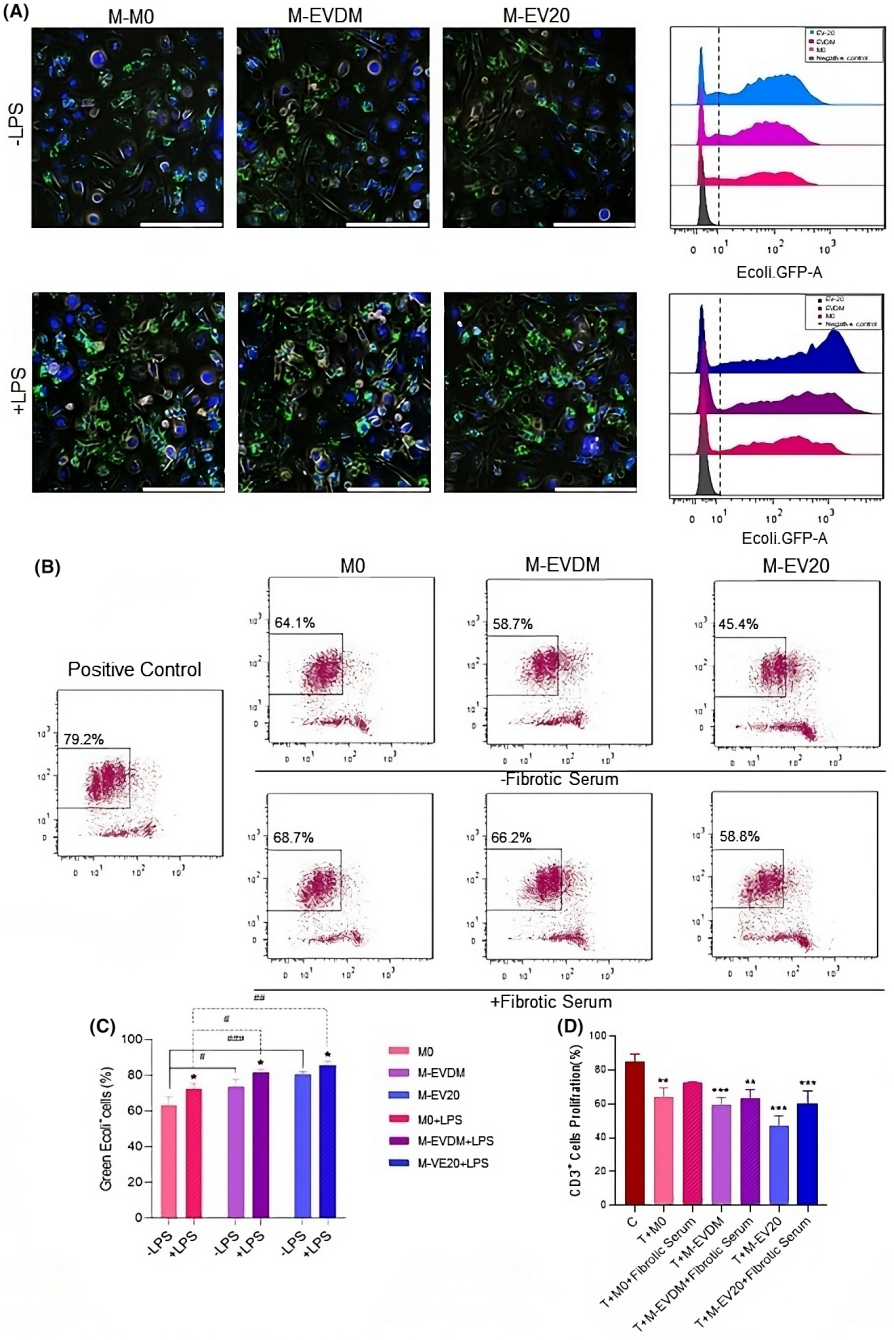
Characterization of anti‐inflammatory properties of EV20K educated macrophages. (A, C) Comparison of the phagocytic activity of differentiated macrophages after incubation with *GFP‐labelled E.coli* in presence or absence of LPS by fluorescent microscopy and flow cytometry. (B, D) The proliferation rates of CFSE‐labelled CD3^+^ lymphocytes in the presence of M0, M‐EVDM and M‐EV20 macrophages (lymphocyte to macrophage ratio of 2:1) with or without serum of mice with CCl4‐induced liver damage, were analysed after 5 days by flow cytometry. Column bars represent the mean ± SD of three independent experiments with macrophages from three different healthy donors. Statistically significant differences were determined by One and two‐way ANOVA followed by Tukey's post hoc test (*p ≤ 0.05, **p ≤ 0.01, ***p ≤ 0.001). The # sign is equivalent to * and was used to avoid confusing the reader (#*p* < 0.05, ##*p* < 0.01, ###*p* < 0.001).

Activated macrophages are generally characterized by their high phagocytosis capacity. We evaluated this parameter in the presence of *Green E.coli* bacteria, with or without LPS stimulation by using flow cytometry and fluorescent microscopy. Following exposure to EVs, M‐EV20 macrophages at 10 μg/mL displayed a notably heightened phagocytic capability as compared to M0 macrophages (M0: 65.5 ± 5.2% vs M‐EVDM: 73.5 ± 4.09% vs M‐EV20: 80.56 ± 1.35%; *p* ≤ 0.001). Co‐stimulation with LPS also resulted in a robust phagocytic capacity in all groups; (M0: 72.7 ± 2.84% vs M‐EVDM: 81.6 ± 1.50% vs M‐EV20: 85.50 ± 2.19%; *p* ≤ 0.01), with a statistically significant (*p* ≤ 0.05) increase in M‐EV20 macrophages after LPS treatment as compared to the M‐EV20 without LPS (Figure 4C).

### 3.5 EV20K maintains its inhibitory effect on the proliferation of allogeneic CD3 ^+^ T cells in fibrotic conditions

The plasticity of macrophages is extremely high as they can change and adapt their characteristics in response to alterations in their environment. Since chronic hepatic damage results in a dramatical change in serum components that may affect the characteristics of macrophages once infused into an animal model of liver disease. To mimic the potential changes in the characteristics of polarized macrophages in response to an in vivo microenvironment, cell‐to‐cell interactions between macrophages and adaptive (T cells) immune cells were assessed in mixed lymphocyte reaction (MLR) in the absence or presence of serum from mice previously treated with CCl_4_ for 5 weeks. Proliferative capacity of allogeneic T cells was tested after 5 days of exposure to M‐EV20, M0 and M‐EVDM macrophages. CD3^+^ T cells activation and proliferation were assessed by flow cytometric analysis. As compared to the positive control, the percentage of proliferating T cells was lower in the three groups of macrophages and this reduction was greater in the M‐EV20 treated group (C: 85.06 ± 4.59% vs M0: 64.13 ± 5.41% vs M‐EVDM: 59.36 ± 4.60% vs M‐EV20: 47.16 ± 5.86%; *p* ≤ 0.001). In addition, while adding fibrotic serum increased the proliferation capacity of T cells in all groups, macrophages showed a stable functional phenotype and exerted their inhibitory effects in comparison to the positive control (C: 85.06 ± 4.59% vs M0: 72.26 ± 0.86% vs M‐EVDM: 63.1 ± 5.61% vs M‐EV20: 60.43 ± 7.40%; *p* ≤ 0.001) (Figure 4B,D). Such results confirmed the preservation of anti‐inflammatory macrophagic phenotype even in the presence of proinflammatory stimuli (Figure 4B,D).

### 3.6 Differentiated macrophages regulated the activation features of LX2 stellate cells

**FIGURE 5:**
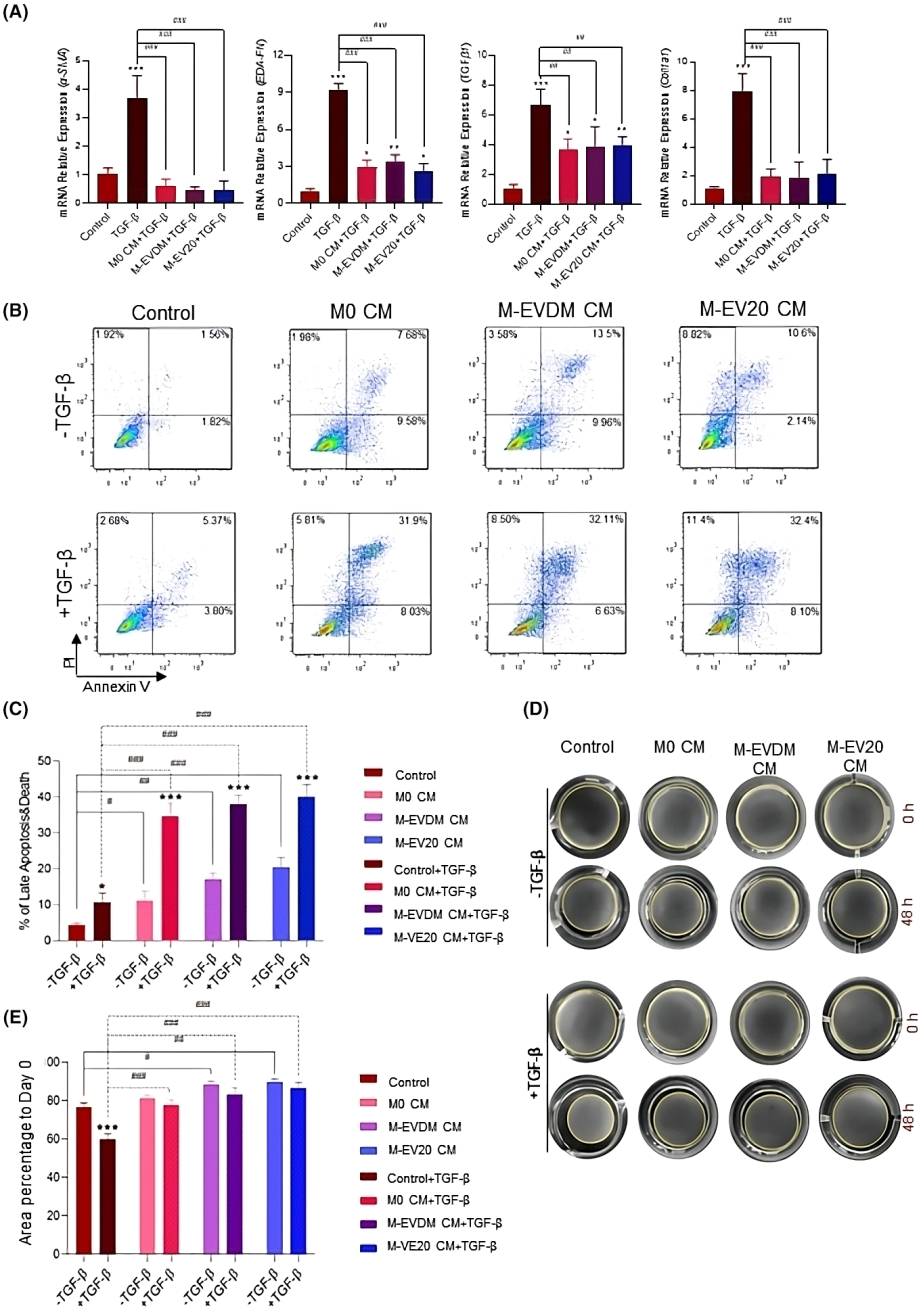
Characterization of anti‐fibrotic properties of EV20K educated macrophages. (A) Evaluating the gene expression pattern of *α‐SMA, FN, TGF‐β* and *Col1α1* in Control (non‐activated LX2 stellate cells), TGF‐β‐activated LX2 stellate cells, and LX2 stellate cells treated with TGF‐β and CM of M0, M‐EVDM, M‐EV20 macrophages. (B, C) The percentage of apoptosis in LX2 stellate cells incubated with M0, M‐EVDM, M‐EV20 macrophages' CM was evaluated by using Annexin V/PI test and flow cytometry. (D, E) LX‐2 stellate cells' contractile activity after treatment with CM of M0, M‐EVDM, M‐EV20 macrophages was quantified by ImageJ software. Column bars represent the mean ± SD of three independent experiments with macrophages from three different healthy donors. Statistically significant differences using Two‐way analysis of variance followed by Tukey's post hoc test (*p ≤ 0.05, **p ≤ 0.01, ***p ≤ 0.001) compared to control group (^#^ *p* ≤ 0.05, ^##^ *p* ≤ 0.01, ^###^ *p* ≤ 0.001).

The macrophage population contributes to the regulation of fibrosis by inhibiting the activation and proliferation of HSCs and downregulating the transcriptional expression of *α‐SMA* and collagen synthesis related genes. In order to evaluate the anti‐fibrotic properties of macrophages differentiated under our experimental conditions, and the viability and gene expression pattern of LX2 cells (activated or no not by TGF‐β) were examined in the presence of CM recovered from M0 control, EVDM and EV20 culture groups. Our analyses revealed the significant reduced expression of *α‐SMA, Fibronectin 1, TGF‐β* and *Collagen type1* in activated LX2 cells after treatment with macrophage CM (Figure 5A). In addition, qualitative live/dead (Figure S1) and quantitative flow cytometric analysis demonstrated macrophages CM significantly induce apoptosis in both non‐activated (C: 4.29 ± 0.54% vs M0: 11.12 ± 2.68% vs M‐EVDM: 17.08 ± 1.69% vs M‐EV20: 20.45 ± 2.75%; *p* ≤ 0.001) and activated LX2 cells (C: 10.67 ± 2.65% vs M0: 34.74 ± 3.59% vs M‐EVDM: 38.09 ± 2.40% vs M‐EV20: 40.08 ± 3.49%; *p* ≤ 0.001) (Figure 5B,C). These results support the inhibitory effect of macrophages on the activation of LX2 stellate cells by preventing the alteration of their cellular phenotype and by inducing cell death.

### 3.7 TGF‐β1‐induced contractility of LX2 is inhibited by macrophage CM

HSC activation exhibits many cellular features including cell contractility. Therefore, we analysed the contractility of LX2 cells after their incubation with macrophage CM and further TGF‐β1 stimulation, by using a collagen gel contraction assay. The validation of experiments was supported by the demonstration of significant higher contraction of the gels containing LX2 cells treated with TGF‐β1 as compared to non‐ TGF‐β1 treated LX2 groups. Treatment with CM from macrophage populations significantly decreased the capacity of LX2 cells to contract the collagen gel in both TGF‐β treated and non‐treated groups, suggesting that differentiated macrophages in all groups presented such inhibitory effects on LX2 cells (Figure 5D,E).

## 4 DISCUSSION

It is increasingly known that eliminating the agents causing liver damage can allow partial reversibility of liver fibrosis, which results in intrinsic liver regeneration.^32^ Several studies have demonstrated that macrophages play an important role in repairing and restoring fibrotic liver conditions thanks to their paracrine and chemotactic functions.^3^, ^33^, ^34^ Macrophage therapy reversed liver damage induced by CCL4 by reducing the level of myofibroblasts and increasing available anti‐inflammatory cytokines.^35^ Furthermore, macrophages have been shown to be effective in removing fibrotic scars by producing matrix metalloproteases and phagocytosis.^36^ Macrophages also directly stimulated the differentiation of liver progenitor cells into mature hepatocytes via an activation of TWEAK/FN14 signalling pathways.^37^ Those promising outcomes observed in vivo have paved the way for the initiation of clinical trials utilizing autologous macrophages within an anti‐inflammatory phenotype to treat patients with liver cirrhosis and further demonstration of the safety and feasibility of such treatment approach.^38^, ^39^ However, the use of the patient's own cells in cell therapy approaches has encountered a number of challenges and issues, such as the difficulty to recover an adequate number of cells as well as the impact of recipient physiological conditions and genetic background on their quality, proliferation and differentiation capacity. Seeking to overcome these barriers, the use of allogeneic cells from a donor with a compatible tissue type is actively under development.^40^, ^41^ In recent years, birth related products, such as UCB, placenta and umbilical cord tissue have attracted great attention as valuable sources of allogeneic cells. UCB is a non‐invasive and accessible source that provides a sufficient number of immune cells with high proliferation and differentiation capacity.^42^, ^43^ In our previous work, we demonstrated that UCB can serve as a reliable alternative source of monocytes, which can be effectively polarized into both proinflammatory and anti‐inflammatory functional macrophages.^28^ In the current study, we investigated the potential of EVs derived from WJ‐MSCs in inducing the polarization of UCB‐derived monocytes into functional anti‐inflammatory and anti‐fibrotic macrophages as previously documented in vitro.^44^, ^45^ MSCs are known to produce a large panel of potent bioactive molecules including growth factors, cytokines and chemokines, that regulate the behaviour and functions of macrophages.^46^, ^47^

The crucial aspects of current challenges in the broad clinical application of EVs are considerable difficulties in their efficient, reproducible and scalable isolation methods. While using ultracentrifugation (110,000 *g*) is the gold standard method for isolating EVs, it has many practical drawbacks, including limited availability of ultracentrifuges in a few labs, the potential damage to EVs, time/cost constraints and heterogeneity of populations because of the overlaps in density or size of the different EV types.^48^, ^49^ Thus, subpopulations of EVs centrifuged at lower speeds (<110,000 *g*) are more accessible and have been recommended in other studies.^50^, ^51^ In this study, we isolated 2 subpopulations of EVs (EV20K and EV110K) using high‐speed centrifugation and ultracentrifugation methods, respectively, and characterized them in accordance with the MISEV criteria.^49^ Subsequently, we assessed the differentiation and maturation of UCB monocytes following exposure to different concentrations of WJ‐MSC‐EVs. Gene expression analysed data revealed that EV110K at a concentration of 50 μg/mL caused a significant increase in proinflammatory genes (*IL‐6, IL‐1β, TNF‐α*) in differentiated macrophages, while also upregulated specific anti‐inflammatory genes. In contrast, EV20K at a concentration of 10 μg/mL, significantly enhanced the expression of genes related to anti‐inflammatory cytokines (*TGF‐β, IL‐10*), scavenger receptors (*MRC1, CD163, CD169*), ECM turnover (*MMP‐1, MMP‐2*) and liver‐specific growth factors (*HGF, TWEAK*) in treated macrophages with no impact on the expression of proinflammatory markers that was also aligned with enhanced phagocytic, tissue repair and immunosuppressive abilities in those macrophages. Variations in protein and RNA content and composition may explain the difference between EV20K and EV110K in stimulating an anti‐inflammatory phenotype in those derived macrophages. Studies have shown that large MSC‐derived EVs contain more proteins by volume as compared to small EVs.^50^ Furthermore, large MSC‐derived EVs contain higher levels of miRNAs such as miR‐21, −24, −214 and −17, which play crucial roles in regulating inflammation and promoting anti‐inflammatory responses.^51^ In our previous study, we conducted a comprehensive quantitative proteomic analysis on MSC‐derived EVs obtained using high‐speed centrifugation (HS), ultracentrifugation (UC) and sucrose cushion (SU). Our results showed a higher abundance of proteins involved in translation and metabolism pathways, such as oxidative phosphorylation, in the HS group compared to the SU and UC groups. During M2 macrophage polarization, a significant occurrence involves a metabolic transition towards oxidative phosphorylation, which is more efficient in generating ATP compared to glycolysis.^52^ On the other hand, the HS group also showed a lower abundance of proteins involved in inflammatory responses and cell death in comparison to other groups.

Proteomic examination of the content of EVs also revealed an elevated expression of TGF‐β in EV20K that is aligned with its pivotal role in regulating macrophage function. The biological activity of TGF‐β is dose‐dependent and any changes in its concentrations may induce different effects. It promotes the differentiation of non‐activated macrophages into an M2‐like phenotype in a dose‐dependent manner.^53^ TGF‐β‐stimulated macrophages secrete higher levels of the anti‐inflammatory cytokine IL‐10, exhibit increased expression of the mannose receptor CD206 and display robust pro‐phagocytic activity. Furthermore, TGF‐β dampens the expression of proinflammatory cytokines such as TNF‐α and IL‐12 in macrophages.^54^ Indeed, the ability of MSCs to induce macrophage polarization towards an anti‐inflammatory phenotype has been shown to be hampered in the presence of TGF‐β receptor inhibitor, which suggests the critical role of this growth factor in promoting such specific differentiation process.^45^ Finally, our previous studies showed the efficiency and remarkable performance of the EVs isolated by high‐speed centrifugation (20,000 *g*) in promoting tissue repair through inhibition of excessive fibrosis and inflammation modulation.^53^, ^54^, ^55^

Our results revealed a similarity between M‐CSF and EVDM groups in terms of gene and protein expression pattern, which confirmed that MSCs secrete high amounts of soluble proteins and growth factors into the surrounding environment, which can have a direct effect on target cells independently of EVs.^56^, ^57^ Several studies have shown that inflammatory conditions potentiate the differentiation and polarization of macrophages into anti‐inflammatory phenotype, which will lead to an increase of their phagocytic capacity which promotes their efficient removal of damaged hepatocytes and HSCs from fibrotic environment.^58^, ^59^, ^60^ Here, we demonstrated that the increased phagocytosis capacity of LPS‐stimulated macrophages confirmed their ability to respond to local stimuli and acquire different phenotypes and functions specifically adjusted to physiological changes. Moreover, EV20K‐treated macrophages produced high levels of IL‐10 and TGF‐β which maintained their inhibitory effect on the T cells' proliferation in response to allogeneic stimuli, including the use of serum from mice with CCl4‐induced livers damage. Evaluation of the gene expression pattern in macrophages treated with the serum from mice with CCl4 has revealed the higher expression levels of anti‐fibrotic (MMP8, MMP9), anti‐inflammatory (IL‐10) and angiogenic (VEGF) factors, in parallel to the diminished expression of IL‐6, TNF‐α and INOS.^35^ Overall, these results indicate that the bioactive factors present in the serum of these mice change the characteristics of macrophages towards an M2 phenotype, associated with anti‐inflammatory and tissue repair properties. One of the critical roles that macrophages play in the regulation and resolution of fibrotic processes is the inhibition of HSC activation or the induction of their apoptosis.^61^, ^62^ The results of our study provided compelling evidence that CM derived from differentiated macrophages upon EV20K treatment, effectively hinders the proliferation and activation of LX2 stellate cells by inducing a significant reduction in the gene expression of *α‐SMA* and other relevant matrix proteins in activated myofibroblasts. Furthermore, we demonstrated that the CM of macrophages had a profound and significant impact on LX2 stellate cells viability and function. Indeed, our data revealed a substantial reduction in the contractility properties and triggered apoptosis in both quiescent and activated LX2 cells which is aligned with previous data recovered from co‐cultures of human macrophages and myofibroblasts.^63^, ^64^

In summary, our study unveils a groundbreaking potential in the realm of cell‐free therapy targeting liver fibrosis. The specific subset of EVs derived from WJ‐MSCs as an off‐the‐shelf and GMP‐compatible product (EV20K) might provide a feasible strategy to educate the UCB monocytes into a novel and distinct subset of alternatively activated macrophages. Educated macrophages exhibited not only novel characteristics, but also unique expression profiles of cell surface molecules. Both genetic and protein changes demonstrated a comprehensive alteration in their molecular signature and functional abilities including higher phagocytic activity, which is needed for an effective clearing and restoration of damaged fibrotic tissues. By acquiring both immunoregulatory and anti‐fibrotic properties, those educated macrophages may considerably represent a plausible strategy for the design and development of such advanced cell‐based approach for the treatment of liver fibrosis.

## AUTHOR CONTRIBUTIONS

**Shukoofeh Torabi:** Conceptualization (equal); data curation (equal); investigation (equal); methodology (equal); software (equal); writing – original draft (equal). **Morteza Zarrabi:** Formal analysis (equal); validation (equal); writing – review and editing (equal). **Faezeh Shekari:** Formal analysis (equal); validation (equal); writing – review and editing (equal). **Hedie Poorkazem:** Formal analysis (equal); validation (equal); writing – review and editing (equal). **Majid Lotfinia:** Formal analysis (equal); validation (equal); writing – review and editing (equal). **Stefan Bencina:** Formal analysis (equal); validation (equal); writing – review and editing (equal). **Roberto Gramignoli:** Conceptualization (equal); supervision (equal); writing – review and editing (equal). **Moustapha Hassan:** Conceptualization (equal); supervision (equal); writing – review and editing (equal). **Mustapha Najimi:** Conceptualization (equal); supervision (equal); writing – review and editing (equal). **Massoud Vosough:** Conceptualization (equal); funding acquisition (equal); project administration (equal); supervision (equal); writing – review and editing (equal).

## FUNDING INFORMATION

This study was funded by grants from Bahar Tashkhis Teb Co (BT‐9906), Royan Institute (R1399‐99000082) and Royan Stem Cell Technology (RST‐99000082) to MV, and a grant from Åke Wiberg Stiftelse (nr.M20‐0192) to RG.

## CONFLICT OF INTEREST STATEMENT

Nothing to declare.

## Supporting information


Figure S1:

Table S1:

Data S1.

## Data Availability

Data available on request from the authors.
