# Supplementary figures and images for: Wharton's Jelly mesenchymal stem cell‐derived extracellular vesicles induce liver fibrosis‐resolving phenotype in alternatively activated macrophages
Source: J Cell Mol Med. 2024 Sep 17;28(18):e18507. doi: 10.1111/jcmm.18507 (PMC11407755; doi:10.1111/jcmm.18507)

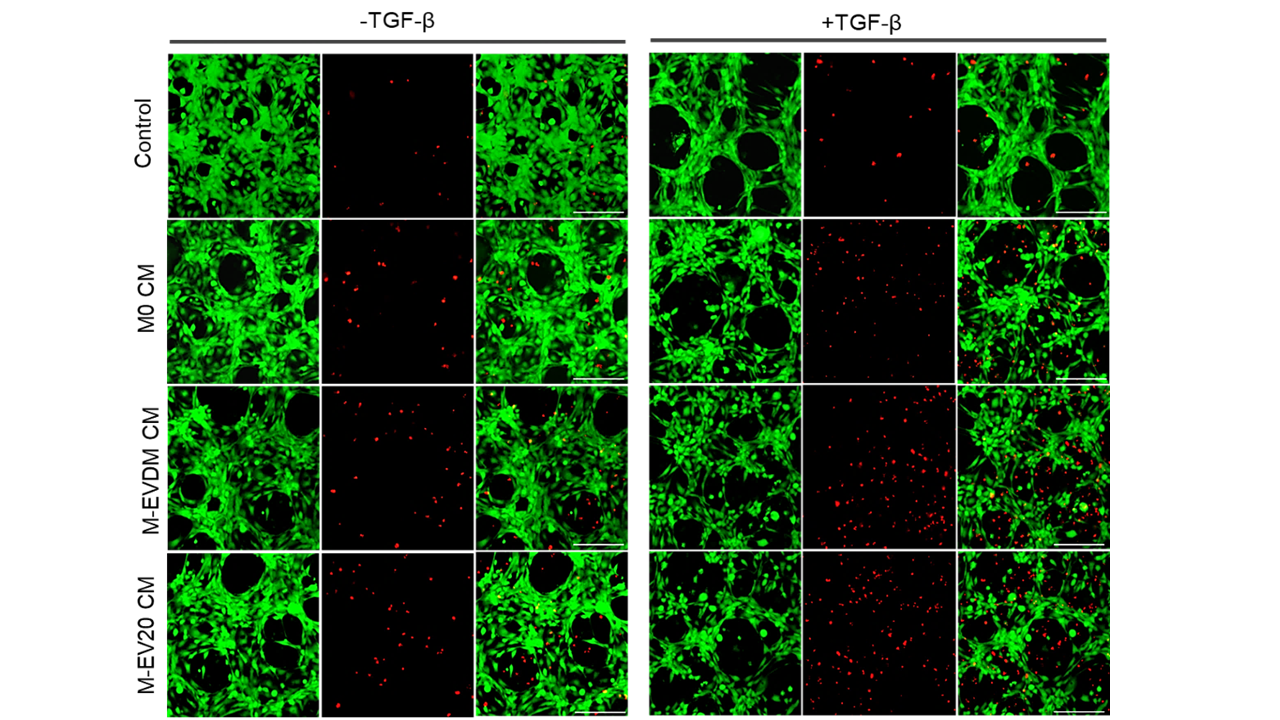

Supplement: Supplementary file 1 — Figure S1: [file JCMM-28-e18507-s002.tif]
