# Supplementary material for: Wharton's Jelly mesenchymal stem cell‐derived extracellular vesicles induce liver fibrosis‐resolving phenotype in alternatively activated macrophages
Source: J Cell Mol Med. 2024 Sep 17;28(18):e18507. doi: 10.1111/jcmm.18507 (PMC11407755; doi:10.1111/jcmm.18507)
Supplement: Supplementary file 2 — Table S1: [file JCMM-28-e18507-s001.docx]

| **Product Size (bp)** | **Annealing Temperature (°C)** | **Sequence 5′ → 3′** | **Name** |
| --- | --- | --- | --- |
| 176 | 60 | F:TGCAAAACCAAACCACAAGA  R: TCTCGGAGATCTCGAAGCAT | IL-10 |
| 144 | 60 | F:GAAACCCACAACGAAATCTATGAC  R: TAACTTGAGCCTCAGCAGAC | TGF-β1 |
| 127 | 60 | F: TTTGTCAACTTGAGTCCCTTCAC  R: TCCCGCTACACTTGTTTTCAC | CD163 |
| 125 | 60 | F: CAGACACGATCCGACCCTTC  R: GTCTCCGCTTCATGCCATTG | MRC1 |
| 99 | 60 | F: CCACTAGGGCTGATACTGGCT  R: GAGGCGGGTGGTTGACTAC | SIGLEC1  (CD169) |
| 180 | 60 | F: CTG TCCTGCGTGTTGAAAGA  R: TTCTGCTTGAGAGGTGCTGA | IL-1β |
| 212 | 60 | F: GGGCCTGTACCTCATCTA  R: AGACCCCTCCCAGATAGATG | TNF-α |
| 180 | 60 | F: AGGAGACTTGCCTGGTGAAA  R: CAGGGGTGGTTATTGCATCT | IL-6 |
| 82 | 60 | F: AAAATTACACGCCAGATTTGCC  R: GGTGTGACATTACTCCAGAGTTG | MMP1 |
| 202 | 60 | F: ATTGTATTTGATGGCATCGCTC  R; ATTCATTCCCTGCAAAGAACAC | MMP2 |
| 103 | 57 | F: TCCAAGGCTCTGAAAAGGGC  R: ATTCAGGCTATCTGGGACCG | TIMP1 |
| 286 | 60 | F: CGCCTTTCCTGAACCGACTA  R: AGGTAGACAGCCTTCCCCTC | TWEAK |
| 261 | 60 | F: ACAGCTTTTTGCCTTCGAGC  R: GCAAGAATTTGTGCCGGTGT | HGF |
| 197 | 60 | F: GGAGCGAGATCCCTCCAAAAT  R: GGCTGTTGTCATACTTCTCATGG | GAPDH |
| 87 | 60 | F: GGAGCGAGATCCCTCCAAAAT  R: GGCTGTTGTCATACTTCTCATGG | ACTA2  (α-SMA) |
| 101 | 60 | F: ACCACATCGAGCGGATCTG  R: TCTGTGACACAGTGGCCATAGG | Fibronectin |
| 133 | 60 | F: GCCAAGGGTCTGACTGG  R: CCCATCACACCAGCCTG | Collagen1α1 |

**Supplementary Table S1:** List of the primers used in the study
