# Supplementary material for: Wharton's Jelly mesenchymal stem cell‐derived extracellular vesicles induce liver fibrosis‐resolving phenotype in alternatively activated macrophages
Source: J Cell Mol Med. 2024 Sep 17;28(18):e18507. doi: 10.1111/jcmm.18507 (PMC11407755; doi:10.1111/jcmm.18507)
Supplement: Supplementary file 3 — Data S1. [file JCMM-28-e18507-s003.docx]

**Legend of supplementary figure 1:**

Live/dead assay method to investigate cell compatibility and survival of LX2 cells after treatment with CM of M0, M-EVDM, M-EV20 macrophages in the presence and absence of TGF-β. Scale bar: 200µm
